# The Apoptotic, Oxidative and Histological Changes Induced by Different Diameters of Sphere Gold Nanoparticles (GNPs) with Special Emphasis on the Hepatoprotective Role of Quercetin

**DOI:** 10.34172/apb.2024.014

**Published:** 2023-10-14

**Authors:** Wael A. M. Ghonimi, Foll alnada A. F. Abdelrahman, Gamal A. Salem, Naief Dahran, Shafika A. El sayed

**Affiliations:** ^1^Department of Histology and Cytology, Faculty of Veterinary Medicine, Zagazig University, 44519 Zagazig, Egypt.; ^2^Department of Pharmacology, Faculty of Veterinary Medicine, Zagazig University, 44519 Zagazig, Egypt.; ^3^Department of Anatomy, Faculty of Medicine, University of Jeddah, Jeddah, Saudi Arabia.

**Keywords:** Gold nanoparticle, Apoptosis, Oxidative stress, Nanoparticles, Quercetin, Toxicity, GNPs

## Abstract

**Purpose:**

Gold nanoparticles (GNPs) as pharmaceutical and drug delivery tools exhibited harmful effects on human health and other living species. Quercetin (Qur) reveals various pharmacological effects specially antioxidant, anti-inflammatory and antiapoptotic. This study is directed to investigate hepatotoxicity of GNPs, in addition, to assess the impact of Qur in mitigating the toxicological effects of GNPs.

**Methods:**

Groups of rats were treated with or without sphere GNPs (10, 20 and 50 nm) and Qur (200 mg/kg b.wt.). Blood and liver samples from euthanized rats were subjected to biochemical, hematological, histopathological, and immunohistochemical investigations.

**Results:**

In comparison with 20 and 50 nm treated groups, the 10 nm GNPs significantly increased serum hepatic enzymes, aspartate aminotransferase (AST), alanine transaminase (ALT), alkaline phosphatase (ALP) and bilirubin. These 10 nm GNPs were associated with oxidative stress and markedly decreased antioxidant enzymes: catalase (CAT), glutathione peroxidase (GPX) and superoxide dismutase (SOD). Immunohistochemically, 10 nm GNPs expressed intense positive signals in nuclei of hepatocytes when stained with anti-caspase-3 antibody confirming extensive apoptosis. Pre-cotreatment with Qur decreased all tested hepatic enzymes and increased serum level of antioxidant enzymes compared to 10 nm GNPs. Qur treatment strongly exhibited anti-Ki67 antibody (proliferative marker) indicating high proliferation of hepatic parenchyma. Histopathologically, 10 nm GNPs revealed diffuse hydropic degenerations, severe sinusoidal congestion, coagulative necrosis, sever steatosis and diffuse hemosiderosis within the hepatic parenchyma. Qur treatment ameliorated most of these pathological effects.

**Conclusion:**

The smaller diameters of GNPs induce potential oxidative stress, cytotoxic, and apoptotic effects in hepatic tissues rather than larger ones. In addition, Qur demonstrated a significant prophylactic role against hepatotoxicity of GNPs.

## Introduction

 Nanoparticles (NPs) are considered the basic unit of the nanotechnology because of their small size, ranging from 1 to 100 nm in diameter. The most fundamental characteristic of NPs is their various and small sizes, that makes them distinctive and flexible in all fields. Therefore, they are widely used for many different purposes, including medical therapies, cosmetics, optical devices, biological labeling, clothing, and cancer treatment.^1,2^ Additionally, some NPs can enter, cross the physiological barriers, and travel via the circulatory system to all internal organs, causing a risk for both human and animal health.^3^ NPs of small sizes are more hazardous as they can produce oxidative stress and create free radicals, which can injure & affect tissues, cells, and macromolecules.^4^

 Metal oxide NPs are a part of the family of nanomaterials that are generated using gold, copper, silver, magnesium, zinc, titanium & alginate. Recently, metal oxide NPs have attracted a lot of attention due to their potential usage in domestic & industrial applications such as nanoelectronics, nanosensors, optoelectronics, catalysis, nanodevices & information’s storage.^5-7^ Moreover, metal oxide NPs retention in the environment and food chain is high and the continuous exposure to them may affect human health.^8^

 Gold nanoparticles (GNPs) are one of the earliest metals ever discovered, with a history of research and application dating back at least a thousand years. The first documented records of colloidal gold can be found in the treatises that are written in the fourth and fifth centuries by Arabian, Indian & Chinese scholars. During the Middle Ages, colloidal gold was explored and utilized in alchemist labs all over Europe. Because of its small size and high flexibility across a wide range of fields, as well as its biocompatibility and lack of cytotoxicity, GNPs have attracted considerable interest and has an advantage over other metal NPs. In addition, internally, they are smaller about 100 to 1000 times than human cells and due to their chemical inertness, it has been employed internally in humans for the past 50 years.^9^

 There are many subtypes of GNPs based on the size, shape, and physical properties; gold nanorods, nanospheres, nanocages, SERS; Surface-Enhanced Raman Scattering nanoparticles & nanoshells.^2^

 GNPs are classified as one of the metallic NPs that have a wide range of uses in pharmaceuticals, biosensing medications, in addition to drugs, genes & protein delivery.^10^ GNPs in medical applications are utilized to treat a variety of diseases, such as cancers of the lungs & liver.^11^ Where, greater concentrations of GNPs are appeared to have strongly anticancer activity against lung & liver cancer cells.^12^ Some vitro studies clarified that chloroquine-gold nanoparticle conjugates (GNP-Chl) inhibiting the growth of breast cancer cells.^13^ In addition,GNPs have been employed in imaging, therapeutic, and diagnostic systems, and can be developed into bigger structures as polymeric NPs or drug-loaded liposomes. These encapsulated GNPs have specific uses in the biomedical fields such as theranostics.^14^ Also, GNPs were used in Chemotherapy but its potential is limited in many cases.^15^ Furthermore, GNPs have been utilized in many other applications such as protein assay,^16^ immunoassay,^17^ capillary electrophoresis,^18^ time-of-flight secondary ion mass spectrometry,^19^ and detection of cancer cells.^20,21^

 The cytotoxicity of GNPs is mainly depending up on their size and concentration. The most recent in vitro investigation clarified that the very minute Au-NPs cause different degrees of necrosis in the cells because of their induction of oxidative stress & mitochondrial damage, while the large particles are less harmful.^22^

 Quercetin (Qur) is one of the main flavonoids in the human diet that has considerable pharmacological effects. It can be found in a variety of fruits (apple, grape, strawberry & raspberry), and vegetables (tomato, onion, green bean & lettuce), leaves, seeds and grains. It has a bitter flavor and is utilized as an ingredient in the dietary supplements, drinks, and foods.^23^

 Qur is a well-known antioxidant, anti-inflammatory/anti-allergy agent, anticancer, antidiabetic, antiarthritic, antimicrobial, antiviral, angioprotective, antiobesity, gastroprotective, mood modulator and immunomodulatory.^23-25^ Moreover, numerous investigations have clarified other pharmacological actions of Qur, as anti-proliferative, anti-ageing, anti-angiogenic, renoprotective and hepatoprotective effect.^26-29^ So, as a result of these properties, the Qur is considered as an important therapeutic agent for the prevention & treatment of several diseases induced by oxidative stress and the release of pro-inflammatory materials in the body.^23^

 Our investigation aims to clarify the possible hepatotoxicity induced after the intra-peritoneal injections of different diameters of sphere GNPs, 10, 20 and 50 nm of mature male albino rats with special emphasis on the most toxicological diameter of GNPs. And also, this study was directed to evaluate the possible functional hepatoprotective role of Qur against the hepatotoxicity of GNPs.

## Materials and Methods

###  Animals and housing

 The current investigation had been conducted on 54 apparently healthy mature male albino rats that obtained from the laboratory animal unite, Faculty of Veterinary Medicine, Zagazig University, Zagazig, Egypt. Animal’s weights were 180 ± 20 g and with average three months age.

 Rats were housed in a controlled environment with ideal conditions such as a constant temperature of 20 to 23 °C and a light-dark cycle (14 hours of light and 10 hours of dark was fixed throughout the experiment). Under hygienic conditions, the rats were kept in transparent polypropylene cages, *9 rats/cage*, with free access to water and dry rat pellets feeds. The rats were allowed to acclimatize for a week before starting the experiment for accommodation and avoiding the transportation stress.

###  Supplements 

####  Gold nanoparticles 

 GNPs of different sizes (10, 20 and 50 nm) were purchased from Nano Gate Company, Egypt. All GNPs used in this study were in an aqueous solution at a concentration of 0.01 % and characterized with pH: 8, density: 19.32 g/cm^3^, molecular weight 196.97 and specific surface area 3.394 m^2^/g.

####  Characterization of GNPs by transmission electron microscopy (TEM)

 The mean sizeand shape of these GNPs were evaluated using the Transmission Electron Microscope (JOEL JEM-2100 operating at 200 kV and equipped with Gatan digital camera Erlangshen ES500). The samples were sonicated (Sonication refers to the process of applying sound energy to agitate particles) for 10 minutes. Approximately 5 µL (a drop) of each sample of nanoparticle solution were placed onto carbon-coated copper grids (400 mesh) containing a Formvar resin support ﬁlm (polyvinyl formal resin) and then leave the grid to dry at room temperature for 30 to 45 minutes. TEM images were taken to verify the size and shape distribution of the synthesized GNPs

####  Optical properties

 Absorption spectrum of 10, 20 and 50 nm GNPs were obtained using the Cary 5000 UV-Vis- NIR Spectrophotometer

####  Quercetin (hydrate extra pure)

 Yellow powder was purchased from Nano Gate Company, Egypt. We got Qur as powder with purity 95% by HPLC, Molecular Weight 302.24 g/mol (anhydrous basis), Molecular Formula C15H10O7 and prepare it for using as an aqueous solution according to a dose of 200 mg/kg body weight/day on the water for suspension.

###  Experimental protocol

 The present investigation was performed on mature male albino rats that were grouped randomly (n = 9 rats/ group) for two studies; the first one to evaluate the GNPs toxicity according to the size of NPs, and through one week from the end of the first study, the haematological, biochemical and histopathological toxicity of GNPs was clarified. After that, the second study was started to assessment the protective effect of Qur against the most toxic size of GNPs.

####  Acute toxicity study of GNPs 

 Rats of the first study were subdivided to 4 groups (n = 9 rats/group) and subjected for 7 days to one of the following treatments:

 Group I (G1):rats were kept as a control and fed with a basal diet without treatment neither GNPs nor Qur for 7 days.

 Group II (G2):rats were intraperitoneally injected with 10 nm diameter of sphere GNPs (75 µg/kg body weight of rat) once a day for 7 days.

 Group III (G3):rats were intraperitoneally injected with 20 nm diameter of sphere GNPs (75 µg/kg body weight of rat) once a day for 7 days.

 Group IV (G4): rats were intraperitoneally injected with 50 nm diameter of sphere GNPs (75 µg/kg body weight of rat) once a day for 7 days and the dose was performed according to Orabi et al.^30^

 After 48 hours, rats were euthanized using sevoflurane, liver samples were used for histopathological study and blood samples were collected for hematological, biochemical and oxidative stress markers.

####  Protective study of quercetin 

 Rats of the second study were subjected for 14 days to one of the following treatments:

 Group V (G5): rats were treated with Qur only by oral gavage in a dose 200 mg/kg body weight once a day for 14 days, and the dose was performed according to Abdelhalim et al.^31^

 Group VI (G6): rats were subjected for 14 days; the first seven days treated only with Qur by oral gavage in a dose 200 mg/kg body weight/day. But, for another 7 days, rats were treated with both Qur by oral gavage in a dose 200 mg/kg body weight/day and 10 nm diameter of sphere GNPs intraperitoneally in a dose 75 µg/kg body weight of rat.

 As per the acute toxicity study of different diameters of GNPs, the 10 nm proved to be the most toxic diameter, so it is used for the control positive group in this protective study. In addition, the findings of the control group of the first study were also utilized.

###  Blood sampling 

 After 24 hours of the final dose injection, all rats were denied food for 12–14 hours and thereafter euthanized, and two blood samples from each rat were collected; the first one in EDTA tube containing anticoagulant for hematological test (complete blood count [CBC] concerning on red blood cells [RBCs], white blood cells [WBCs], hemoglobin [Hb], and platelets), and the second sample into sterilized tubes without anticoagulant for serum separation. Centrifugation was carried out at 3000 rpm for 10 minutes to separate the serum, and the serum was conserved at a temperature of −80 °C for various biochemical assessments.

###  Serum biochemical analysis

 To evaluate the liver function, the levels of aspartate aminotransferase (AST), alanine transaminase (ALT), Albumin, Bilirubin (total, direct, indirect) and alkaline phosphatase (ALP) were measured. In addition, to evaluate oxidative stress parameters, the antioxidant enzymes; catalase tests (CAT), glutathione peroxidase test (GPX) and Superoxide dismutase test (SOD) were performed and measured in serum.

###  Histological and histochemical processing

 At the end of experiment, cervical dislocation of rats and for histological analysis, the livers were separated immediately and small pieces from them were taken and fixed in Bouin’s solution firstly then transferred to neutral buffered formalin 10 % for 48 hours. The specimens were then dehydrated in series of ascending grades of ethanol, after that were cleared by xylene and infiltrated in soft melted paraffin in a hot air oven, and embedded in hard paraffin wax forming paraffin blocks. The paraffin blocks were transversely sectioning to the desired thickness of 4-5 µm by using a rotatory microtome and the sections were mounted on a glass slides. The obtained sections were stained with Harris’s hematoxylin & eosin (H&E) for routine histological studies, Mercuric bromophenol bluefor proteins evaluation, Perls’ Prussian blue reaction to detect an excess of iron deposits such as hemosiderin deposits (hemosiderosis) in liver tissue, Blue Masson’s Trichrome for demonstration of collagen fibers and cells cytoplasm, periodic acid–Schiff (PAS) for detection of glycogen & neutral muco-polysaccharides. All these histological and histochemical stains were performed according to Suvarna et al.^32^

###  Anti-caspase 3 & anti-Ki67 immunohistochemical reactivity

 Immunohistochemical staining was performed on 5-μm, formalin-fixed, paraffin-embedded sections using caspase 3 antibodies as a marker of programmed cell death (apoptosis) through the streptavidin–biotin technique. Deparaffinized sections were stained by an indirect immunoperoxidase technique.^33^

 Antigen retrieval was carried out through immersing the sections with 0.1 M citrate buffer solution (pH = 6) for 10 minutes using a microwave (600 W). For eliminating the activity of endogenous peroxidase, sections were incubated with 3% hydrogen peroxide (H2o2) in absolute methanol for 30 minutes at 4 °C. For minimizing nonspecific labeling, sections were incubated with blocking solution that was formed from phosphate-buffered saline (PBS) containing 10% normal goat serum (NGS; life Technology, Germany) and 0.1 % Triton-X-100 (Sigma, Germany). The sections were incubated at 4 °C overnight with the specific primary antibodies diluted in blocking solution using anti-caspase 3 (ab184787), rabbit monoclonal antibodies at 1:1000 dilution or anti-Ki67 (ab15580), rabbit polyclonal, at 1: 200 dilution (Abcam Inc). Sections were incubated with biotin-conjugated secondary antibody at room temperature for 1 hour in PBS containing biotinylated goat anti- rabbit IgG (1:300; Molecular probes). After that, sections were washed in PBS 3 times x 5 minutes for each & subsequently incubated with streptavidin-peroxidase for 30 minutes at approximately 25 °C.

 The final chromogen (streptavidin-biotin complex; immunopositive reactions) were developed & visualized using a 3,3′-diaminobenzidine tetrahydrochloride (DAB)- H2O2 solution, pH 7.0, for 3 min according to the package insert, which produces, an insoluble brown pigment. The sections were then counter stained with Harris haematoxylin and dehydrated with ascending grades of ethanol, cleared with Rothihistol and mounted using Entellan (Merck, Germany).

 The stained sections were photographed by a digital camera (Canon) connected to a light microscope (Zeiss) in the Histology and Cytology department, Zagazig University, Zagazig, Egypt.

###  Statistical analysis

 Results were reported as mean ± SEM (standard error of mean). To assess the influence of the six treatment groups on the different biochemical parameters, one-way analysis of variance (ANOVA) by Fisher tests as post hoc tests were used. The value of *P* < 0.05 was used to indicate statistical significance. All Analyses and charts were done using Statistical Package for Social Sciences version 28.0 (SPSS, IBM Corp., Armonk, NY) and Graph Pad Prism 8.0.2 (GraphPad Software, Inc).

## Results

###  Characterization of the GNPs 

**Figure 1:**
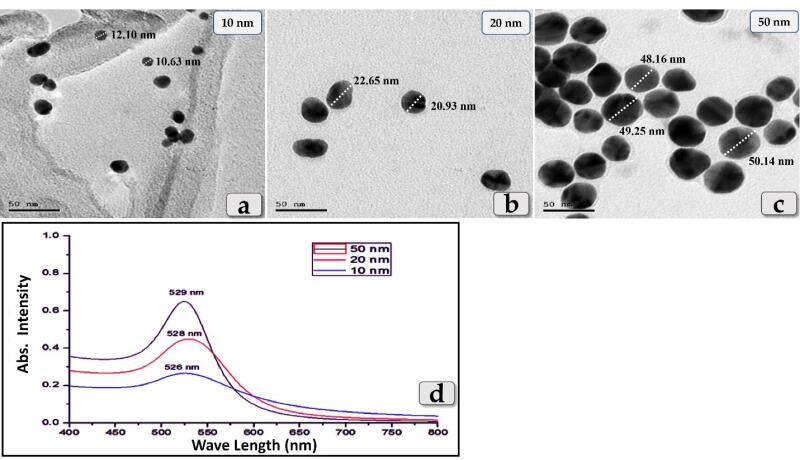
**
(a,b,c) **Transmission electron microscopy (TEM) images showing the size & shape of the GNPs. **(d)** clarifying the wavelength absorption of the three different diameters of GNPs; 10, 20 & 50 nm in the visible region of the optical spectrum (Cary 5000 UV-Vis- NIR Spectrophotometer)

 Some studies have suggested that the biodistribution and toxicity effects of GNPs are size-dependent. So, to investigate whether the size of GNPs affects the liver toxicity, three different diameters of GNPs, 10, 20 & 50 nm were employed in the present study where, the size & shape of the sphere GNPs were evaluated using the transmission electron microscopy (Figure 1a,b,c).In addition, 10, 20 & 50 nm GNPs were exhibited a maximum absorption wavelength around 526 nm, 528 nm & 529 nm respectively where, the peak absorbance wavelength increases with increasing the particle diameter using UV-Vis- NIR Spectrophotometer (Figure 1d).

###  Biochemical analysis

####  Antioxidant enzymes 

**Figure 2:**
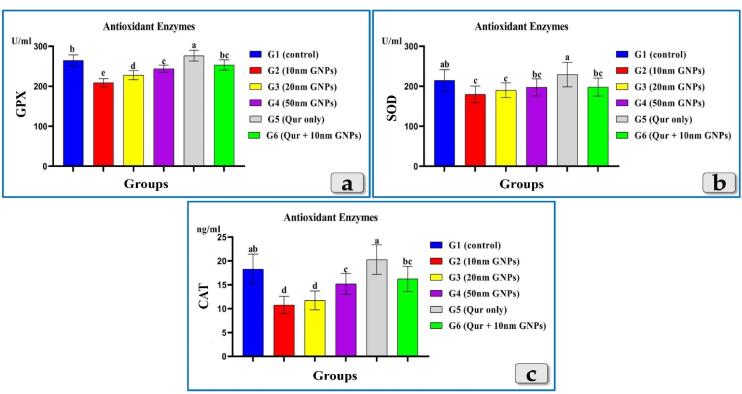
Exhibiting the effect of different diameters of GNPs and quercetin on some antioxidant enzymes; GPX, SOD & CAT for all experimental groups. According to Fisher test, the different letters (a–e) are significantly different between treatments at the *P*< 0.05 level

 All tested antioxidant enzymes; GPX, SOD & CAT clarified a significant decrease in all groups injected with different diameters of GNPs; 10, 20 & 50 nm but the G2 injected with 10 nm was the most affected group and recorded the most decrease for all enzymes levels followed by G3 injected with 20nm and then G4 injected with 50 nm GNPs. Meanwhile, G5 (Qur only) showed elevation in all tested antioxidantenzymes and recorded values near to the control group. But, G6 (Qur & 10 nm GNPs) demonstrated a good value and recorded a significant elevation in all antioxidant enzymes levels compared to G2 (Figure 2).

####  Liver enzymes

**Figure 3:**
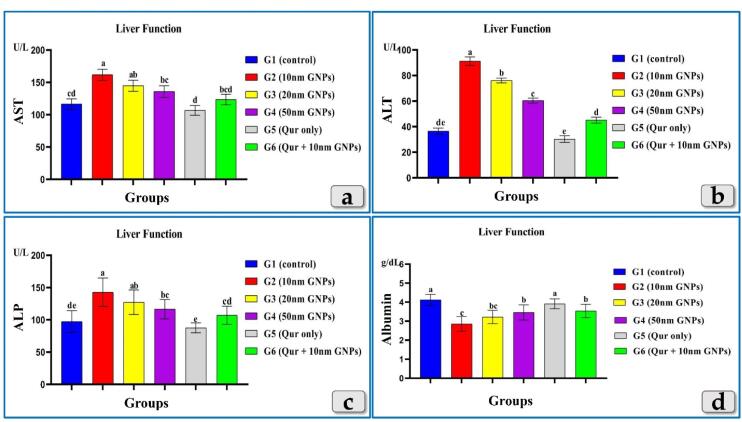
Revealing the effect of different diameters of GNPs and quercetin on some liver functional parameters;AST, ALT, ALP & albumin for all experimental groups. According to Fisher test, the different letters (a–e) are significantly different between treatments at the *P* < 0.05 level

 The tested liver enzymes, AST, ALT & ALP clarified a significant elevation in all groups injected with different diameters of GNPs but the G2 was the most affected group and recorded the highest elevation of AST, ALT & ALP levels followed by G3 then G4. Meanwhile, G5 recorded values near to the control group. In addition, G6 showed a good impact and recorded a significant decrease in AST, ALT & ALP levels compared to G2 (Figure 3).

 Unlike, AST, ALT & ALP, the albumin showed a significant decrease in all injected groups with GNPs. Where, G2 was the most affected group and recorded the highest decrease in albuminlevel followed by G3 then G4. Also, G5 showed level near to the control group value. G6 showed an elevation in albumin level when compared to G2 (Figure 3).

**Figure 4:**
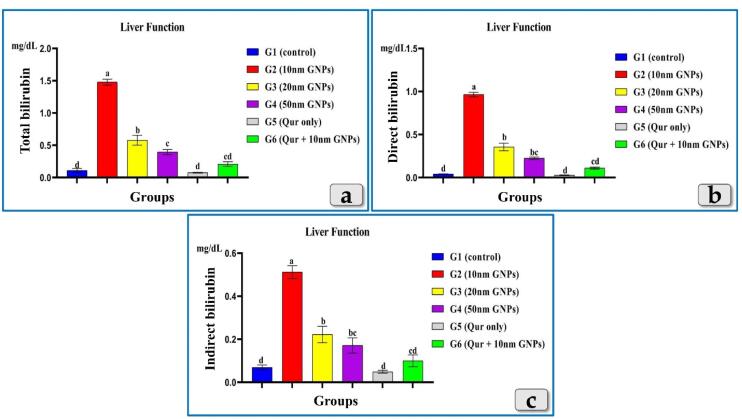
Showing the effect of different diameters of GNPs and quercetin onbilirubin; total, direct & indirectfor all experimental groups. According to Fisher test, the different letters (a–d) are significantly different between treatments at the *P* < 0.05 level

 Resembling AST, ALT & ALP, the bilirubin (total, direct & indirect) showed a significant elevation in all injected groups with GNPs. But G2 was the most affected group and recorded the highest elevation in all bilirubin levels followed by G3 then G4 that recorded the lowest level of the elevation. G5 recorded levels near to the control group value. In addition, G6 demonstrated a good impact and showed a significant decrease in all bilirubin levels compared to G2 (Figure 4).

###  Hematological analysis

**Figure 5:**
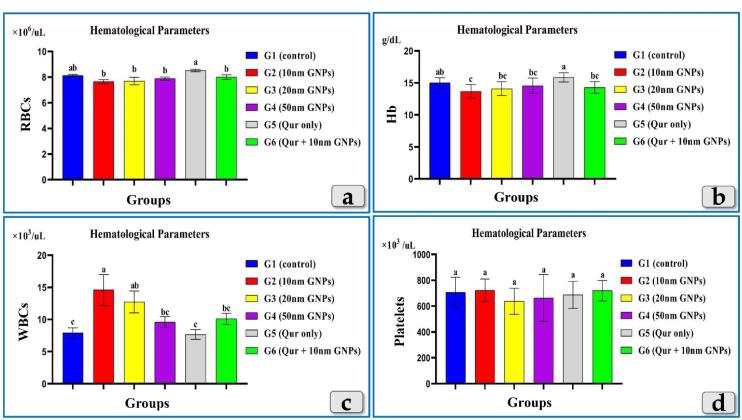
Clarifying the effect of different diameters of GNPs and quercetin on some hematological parameters for all experimental groups; RBCs, HBs, WBCs & Platelets.According to Fisher test, the different letters (a–c) are significantly different between treatments at the *P* < 0.05 level

 The measured hematological parameters; RBCs, HBs, WBCs & Platelets clarified non-significant changes in RBCs & Platelets between all experimental groups (Figure 5) but HBs showed only a significant decrease in G2 injected with 10 nm GNPs but other groups showed non-significant values (Figure 5). Meanwhile, WBCs recorded a significant elevation in G2 & G3 but G2 was the most affected group that recorded the highest elevation between the injected groups. G4 clarified non-significant changes. But, G5 and G6 recorded good values near to the control group (Figure 5).

###  Histopathological analysis

**Figure 6:**
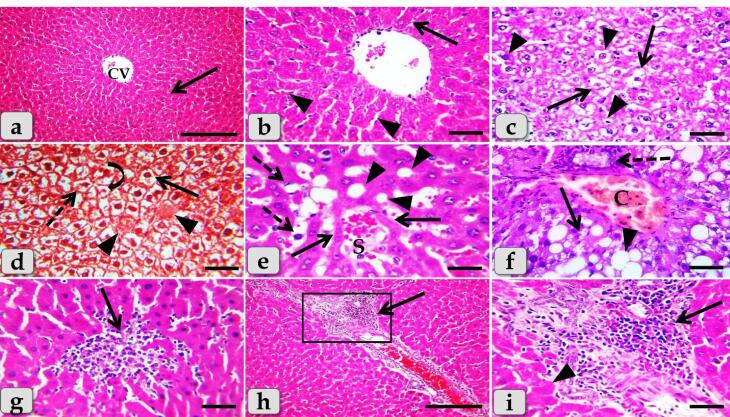
Photomicrographs of the liver of G1 (a& b) control group and G2 (c-i) that injected intra peritoneal with GNPs in a dose of 10 nm for 7 days exhibiting, (a) normal, intact hepatic tissue architecture and cellular details (arrow), with normal central vein (CV). (b) Intact hepatic parenchyma with normal hepatocytes of an irregular polygonal shaped cells with single, central spherical nucleus, and intact hepatic cords (arrow), intact hepatic sinusoids with intact lining epithelium (arrow heads). (c) Diffuse cellular swelling characterized with enlarged, swollen hepatocytes, with small pyknotic central located nuclei and pale cytoplasm with eosinophilic depress (arrow), numerous pleomorphic nuclei of hepatocytes (arrowheads). (d) Diffuse hydropic degenerations (ballooning degeneration) with disorganization of the hepatic cords (arrow), beside single coagulative necrotic cells characterized with enlarged cells, hypereosinophilic cytoplasm with nuclear disappearance (arrowheads). Nuclear changes were obvious in the degenerated hepatocytes; karyorrhexis (dashed arrow) and karyolysis (curved arrow). e) Sever sinusoidal dilatation with congestions (S) accompanied with severe pressure atrophy of hepatic cords (arrows), hyper sinusoidal endotheliosis or endothelial hypertrophy; changes in the endothelial lining the hepatic sinusoids combining with swollen endothelial cells and leading to narrowed sinusoidal lumen (dashed arrows), sever steatosis (arrowheads). (f) Sever steatosis; micro vesiculations (arrow) & macro vesiculations (arrowheads), portal congestion (C) and cholangiocytes proliferations (dashed arrows). (g) Intralobular focal necrotic area replaced by mononuclear cells infiltrations (arrow). (h) Severe inflammatory cells infiltrations surrounding severe dilated & congested blood vessels within the portal triad (arrow). (i) Higher magnification of the inset box of figure (panel h) clarifying severe infiltrations of inflammatory cells of mainly lymphocytes within the portal area (arrow) surrounding with numerous coagulative necrotic hepatocytes (arrowhead). Stain: All) H&E, except d) Blue Masson's trichrome. Scale bars: All = 30 µm, except a & h = 200 µm

 The histopathological examination of the mature male albino rat’s liver of the control group (G1) clarified normal and intact homogenous hepatic parenchyma without any abnormalities. The hepatic parenchyma was composed mainly of numerous classic hepatic lobules. Each lobule was bounded centrally with the central vein (Figure 6a) and peripherally with portal triad that housing intact branches of hepatic artery, portal vein, bile duct, nerve and lymph vessel. The main components of each lobule were the hepatocytes that appeared irregular polygonal in shape with single, central, spherical, euchromatic nucleus, however, some bi-nucleated cells were also observed. These hepatocytes were dorsally radiated from the central vein to the lobule periphery forming the hepatic cords or rays. In addition, intact hepatic sinusoids with intact lining epithelium were also appeared circulating among the hepatic cords supplying the hepatocytes (Figure 6b).

 Meanwhile, the histological examination of the liver of G2 that injected intra peritoneal with 10nm GNPs for 7 days showed diffuse cellular swelling due to hydropic degenerations (ballooning degeneration) in the hepatic parenchyma that characterized with enlarged, swollen hepatocytes, with small pyknotic central located nuclei and pale cytoplasm with eosinophilic depress with general disorganization of the hepatic cords (Figure 6c and 6d). Numerous pleomorphic nuclei with variable size, shape and activity within the hepatocytes were observed (Figure 6c). In addition, some nuclear changes were also obvious in the degenerated hepatocytes as karyorrhexis; fragmentation of the nucleus and breakup of the chromatin that distributed irregularly in the cell cytoplasm and karyolysis; complete dissolution of a cell nucleus (Figure 6d). Beside the hydropic degenerated hepatocytes, single coagulative necrotic cells were distinguished and characterized with maintaining of hepatic tissue architecture with loss of cellular details where, the hepatocytes clarified ghostly appearance with enlarged cells, hypereosinophilic cytoplasm with nuclear disappearance (Figure 6d).

 Sever sinusoidal dilatation with sever congestions accompanied with severe pressure atrophy of hepatic cords were noticed with hyper sinusoidal endotheliosis/or endothelial hypertrophy; changes in the endothelial lining the hepatic sinusoids combining with swollen endothelial cells that leading to narrowed sinusoidal lumen (Figure 6e). Sever steatosis; fatty changes, micro & macrovesicular fat droplets aggregations within the hepatocytes cytoplasm of variable shape and size were described beside portal congestion and bile duct with cholangiocytes proliferations (Figure 6f). Intralobular focal necrotic area replaced by mononuclear cells infiltrations (Figure 6g), with sever inflammatory cells infiltrations of mainly lymphocytes surrounding sever dilated & congested blood vessels within the portal triad were identified surrounding with numerous coagulative necrotic hepatocytes(Figure 6h and 6i).

**Figure 7:**
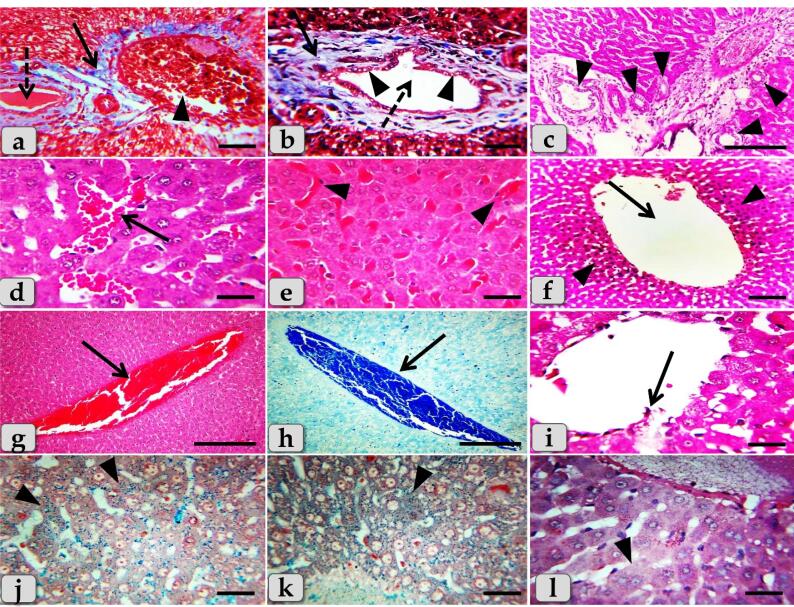
Photomicrographs of the liver of G2 (a-l) that injected intra peritoneal with GNPs in a dose of 10 nm for 7 days clarifying, (a) Sever blood vessels dilatation with severe congestion within the portal triad (arrowhead), sever fibrous connective tissue proliferations of mostly collagen fibers around portal blood vessels and bile duct (arrow), and sever cholestasis; accumulation of bile in the bile ducts (dashed arrow). b) Bile duct hyperplasia (dashed arrow) with sever vacuolations of the ductal lining epithelium (arrowheads), surrounding with sever fibrous connective tissue proliferations of mostly collagen fibers (arrow). c) Sever proliferations of the bile ducts in the portal areas (arrowheads). (d) Sever sinusoidal dilatation with sever congestion in between the hepatocytes (arrow). (e) Sever sinusoidal congestion within the hepatic parenchyma (completely overfilled with RBCs) (arrow heads). (f) Sever dilatation of central vein (arrow) surrounding with diffuse centro-lobular pyknosis of the hepatocytes (arrow heads). (g,h) Sever dilatation and congestion (overfilled with RBCs) of central vein (arrow). (i) Sever degenerations of central vein lining epithelium with sloughing of some lining cells into the lumen (arrow). (j,k) Diffuse hemosiderosis; hemosiderin pigment precipitations within almost hepatic parenchyma (arrowheads). (l) Very pale hepatic parenchyma as a result of diffuse glycogen depletions within the hepatocytes cytoplasm (arrowheads). Stain: All) H&E, except (a,b) Blue Masson's trichrome, (h) Mercuric Bromophenol blue, (j,k) Perls Prussian blue, (l) PAS. Scale bars: All = 30 µm, except c, g& h = 200 µm

 Sever blood vessels dilatation with severe congestion within the portal triad were remarked with increased amount of fibrous connective tissue proliferations of mostly collagen fibers around portal blood vessels and bile duct, with sever cholestasis; over accumulation of bile in the bile ducts (Figure 7a). In addition, bile duct hyperplasia with sever vacuolations of the ductal lining epithelium, surrounding with increased amount of fibrous connective tissue proliferations of mostly collagen fibers were observed (Figure 7b), with sever proliferations of the bile ducts in the portal areas (Figure 7c). Sever sinusoidal dilatation with sever congestion (completely overfilled with RBCs) in between the hepatocytes were noticed (Figure 7d and 7e).

 In some examined sections, the central vein showed severs dilatation surrounding with diffuse centro-lobular pyknosis of the hepatocytes (Figure 7f), with sever congestion (Figure 7g & h), and sever degenerations of its lining epithelium with sloughing of some lining cells into the lumen (Figure 7i). With Perls Prussian blue stain, diffuse hemosiderosis (hemosiderin pigments precipitation) within almost hepatocytes cytoplasm were clarified (Figure 7j and 7k). With PAS stain, very pale hepatic parenchyma was also identified as a result of diffuse glycogen depletions within the hepatocytes cytoplasm (Figure 7l).

**Figure 8:**
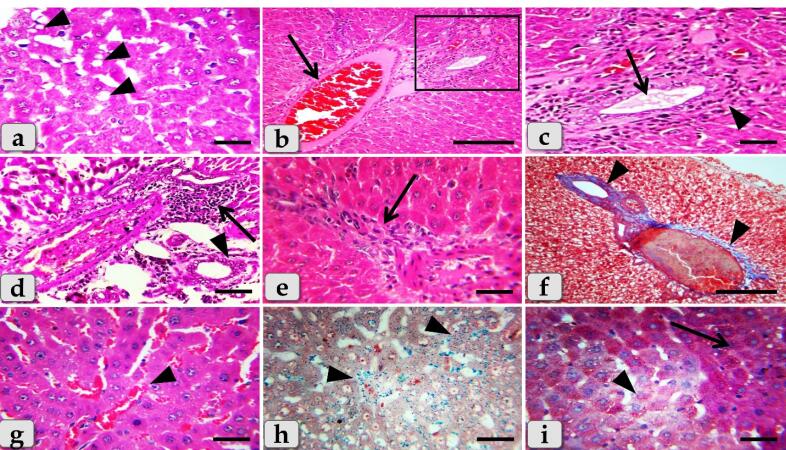
Photomicrographs of the liver of G3 (a-i) that injected intra peritoneal with GNPs in a dose of 20 nm for 7 days revealing, (a) Moderate hydropic degeneration of the hepatocytes (arrowheads). (b) Moderate blood vessels dilatation with moderate congestions in portal triad (arrow). (c) Higher magnification of figure b showing moderate hyperplasia of the bile duct with moderate cholestasis (arrow) with moderate periductal inflammatory cells infiltrations (arrowhead). (d) Moderate inflammatory cells infiltrations in portal triad surrounding portal blood vessels (arrow), with stratification of the bile duct lining epithelium (arrowhead). (e) Moderate inflammatory cells infiltrations in between the hepatocytes (arrow). (f) Moderate fibrous connective tissue proliferations of mostly collagen fibers surrounding the portal blood vessels and bile duct (arrowheads). (g) Moderate sinusoidal dilatation with congestion in between the hepatocytes (arrowhead). (h) Moderate hemosiderosis within the hepatic parenchyma (arrowheads). (i) Small pale patches of hepatocytes with glycogen depletions (arrowhead) surrounding with normal hepatic parenchyma having normal glycogen amount within the hepatocytes cytoplasm (arrow). Stain: All) H&E, except (f) Blue Masson's trichrome, (h) Perls Prussian blue, (i) PAS. Scale bars: All = 30 µm, except b& f = 200 µm

 While, the liver of G3 that injected intraperitoneally with 20 nm GNPs for 7 days revealed moderate hydropic degeneration of the hepatocytes (Figure 8a), moderate blood vessels dilatation and congestion in the portal triad (Figure 8b). And also, moderate bile duct hyperplasia with moderate cholestasis and periductal inflammatory cells infiltrations were observed (Figure 8c). In addition, moderate inflammatory cells infiltrations surrounding the portal blood vessels in the portal triad, with stratification of the bile duct lining epithelium were also demonstrated (Figure 8d). Furthermore, moderate inflammatory cells infiltrations in between the hepatocytes (Figure 8e) with moderate fibrous connective tissue proliferations of mostly collagen fibers surrounding the portal blood vessels and bile duct were also identified (Figure 8f)with moderate sinusoidal dilatation & congestion (Figure 8g). With Perls Prussian blue stain, moderate hemosiderosis within the hepatic parenchyma were clarified (Figure 8h). With PAS stain, small pale patches of hepatic parenchyma with glycogen depletions surrounding with normal hepatic parenchyma having normal glycogen amount within the hepatocytes cytoplasm were distinguished (Figure 8i).

**Figure 9:**
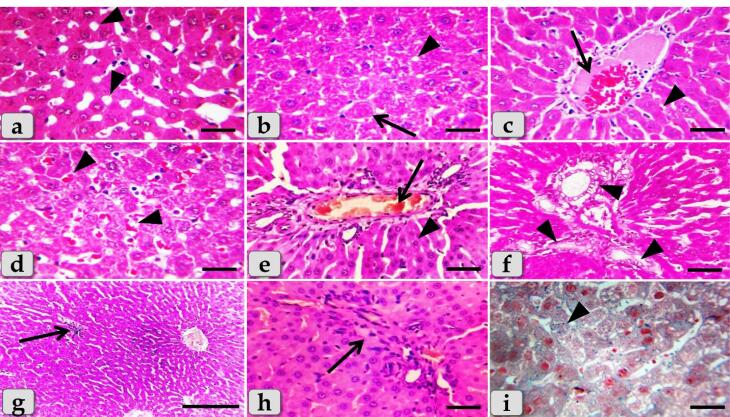
Photomicrographs of the liver of G4 (a-i) that injected intra peritoneal with GNPs in a dose of 50 nm for 7 days showing, (a) Normal, intact hepatic parenchyma but with slightly hydropic degeneration of the hepatocytes (arrowheads). (b) Slightly hydropic degeneration of the hepatocytes (arrowheads) with individual or single coagulative necrotic cell (arrow). (c) Mild dilatation & congestion of central vein (arrow) surrounding with normal organized hepatic cords of normal hepatocytes (arrowhead). (d) Mild sinusoidal dilatations & congestions in between hepatocytes (arrowhead). (e) Mild dilatation & congestion of portal blood vessels (arrow) surrounding with normal organized hepatic parenchyma (arrowhead). (f) Mild dilatation & proliferation of bile duct (arrowhead). (g) Intact hepatic parenchyma with obvious hepatic rays but with mild proliferation of von Kupffer cells in between the hepatocytes (arrow). (h) Mild inflammatory cells infiltrations in between the hepatocytes (arrow). (i) Mild hemosiderosis within the hepatic parenchyma (arrowhead). Stain: All) H&E, except (i) Perls Prussian blue. Scale bars: All = 30 µm, except g = 200 µm

 The liver of G4 that injected intraperitoneally with 50 nm GNPs for 7 days revealed intact hepatic parenchyma but with slightly hydropic degeneration of the hepatocytes (Figure 9a and 9b), with individual coagulative necrotic cell (Figure 9b). In addition, mild congestion of central vein surrounding with normal organized hepatic cords of normal hepatocytes was observed (Figure 9c). Furthermore,mild sinusoidal congestions were clarified (Figure 9d),withmild dilatation & congestion of portal blood vessels (Figure 9e), andmild proliferation of bile duct (Figure 9f). Moreover, intact hepatic parenchyma with obvious hepatic rays were detected but with mildproliferation of von Kupffer cells in between the hepatocytes (Figure 9g). And also,mild inflammatory cells infiltrations were described (Figure 9h) withmild hemosiderosis within the hepatic parenchyma (Figure 9i).

**Figure 10:**
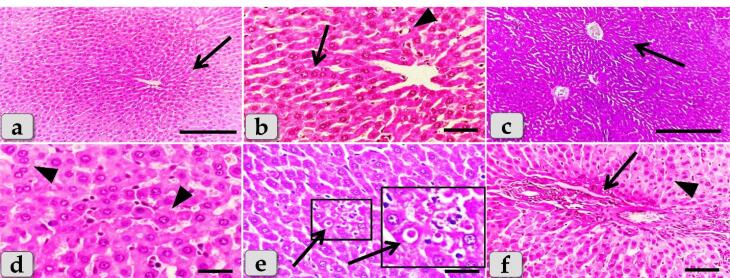
Photomicrographs of the liver of G5 (a,b) that treated with quercetin (Qur) only by oral gavage for 14 days and G6 (c-f) that treated orally with Qur. for along 14 days then injected intra peritoneal with gold NPs in a dose of 10 nm for the last 7 days clarifying, (a) G5 showing normal, intact hepatic parenchyma without any abnormalities (arrow). (b) Higher magnification of figure a showing normal hepatocytes of an irregular polygonal shaped cells with single, central spherical euchromatic nucleus, that normally organized forming intact hepatic cords (arrow), intact hepatic sinusoids with intact lining epithelium were distributing in between the hepatocytes (arrowhead). (c) G6; treated group with Qur & 10 nm GNPs showing normal, intact hepatic parenchyma that appeared looks like normal with normal tissue architecture & cellular details without any abnormalities; intact hepatocytes and vasculature, obvious hepatic cords (arrow). (d) Normal hepatocytes of an irregular polygonal shaped cells with single, central spherical euchromatic nucleus and sometimes binucleated cells were also observed (arrowheads), in addition, individual von Kupffer cells were distributed in between the hepatocytes. (e) In some examined sections, the hepatic parenchyma clarified individual apoptotic cells; Councilman’s bodies apoptotic cell or Councilman hyaline body or apoptotic body surrounded by normal parenchyma where the apoptotic cell is characterized with small sized cell with small dark pyknotic nucleus with central or peripheral position and hypereosinophilic cytoplasm due to housing large acidophilic globules (arrow). Inset box is the higher magnification of the apoptotic cell (arrow). (f) Normal & intact portal blood vessels and bile duct in the portal triad (arrow) surrounding with normal hepatic parenchyma (arrowhead). Stain: All) H&E. Scale bars: All = 30 µm, except a = 200 µm & c = 600 µm

 The liver of G5 that treated with Qur only by oral gavage for 14 days showed normal, intact hepatic parenchyma without any abnormalities (Figure 10a) and the hepatic lobules were filled with normal, intact hepatocytes of an irregular polygonal shaped cells with single, central spherical euchromatic nucleus and normally organized forming intact hepatic cords with intact hepatic sinusoids in between the cords with intact lining epithelium (Figure 10b).

 Moreover, G6 that treated orally with Qur. for along 14 days then injected intra peritoneal with gold NPs in a dose of 10 nm for the last 7 days showed a significant good results when compared with G2 where the hepatic parenchyma was appeared looks like normal with normal tissue architecture & cellular details without any abnormalities; intact hepatocytes, vasculature and obvious hepatic cords (Figure 10c). In addition, thehepatocytes were appeared normal and intact of an irregular polygonal shaped cell with single, central spherical euchromatic nucleus and sometimes binucleated cells were also observed, and also, individual von kupffer cells were distributed in between the hepatocytes (Figure 10d).

 But, in some examined sections,the hepatic parenchyma clarified individual apoptotic cells; Councilman’s bodies apoptotic cell or Councilman hyaline body or apoptotic body surrounded by normal parenchyma where the apoptotic cell is characterized with small sized cell with small dark pyknotic nucleus with central or peripheral position and hypereosinophilic cytoplasm due to housing large acidophilic globules (Figure 10e). And also, normal & intact portal blood vessels and bile duct in the portal triad surrounding with normal hepatic parenchyma were distinguished (Figure 10f). In addition, semi quantitative histopathological lesion scores for all experimental groups were recorded inTable 1.

**Table 1:** Semi-quantitative histopathological lesions score of all experimental groups

	**G1** **(control)**	**G2** **(10nm GNPs)**	**G3** **(20nm GNPs)**	**G4** **(50nm GNPs)**	**G5** **(Qur only)**	**G6** **(Qur+10nm GNPs**)
Hydropic degenerations of hepatocytes with disorganization of hepatic cords within the hepatic parenchyma	**-**	**+++**	**++**	**+**	**-**	**-**
Coagulative necrotic hepatocytes	**-**	**+++**	**++**	**+**	-	+
Sinusoidal dilatation and congestions	**-**	**+++**	**++**	**+**	-	-
Steatosis (fat droplets aggregation within the hepatocytes)	**-**	**+++**	**++**	**+**	-	-
Inflammatory cells infiltrations	**-**	+ + +	+ +	+	-	+
Blood vessels dilatation and congestion within the portal triad	**-**	**+++**	**++**	**+**	-	-
Apoptotic cells	**-**	+ + +	+ +	+	-	+
Proliferation of bile duct	**-**	+ + +	+ +	+	**-**	**-**
Hemosiderosis	**-**	**+++**	**++**	**+**	**-**	**-**
Glycogen depletions	**-**	**+++**	**++**	**+**	**-**	**-**

**Figure 11:**
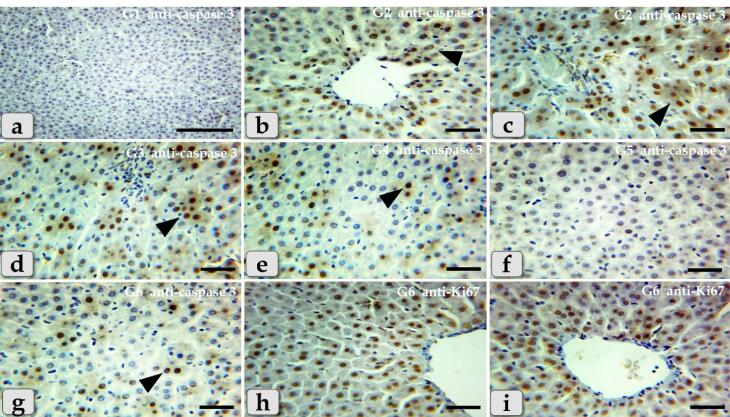
Aphotomicrographs of the immunohistochemical staining of the liver against anti-caspase-3 antibody in G1-6, in addition anti-Ki67 antibody in G6 where, the positive signal is mostly expressed in the nuclei of morphologically identifiable apoptotic cells when stained with anti-caspase-3 antibody but mostly expressed in the proliferated cells when stained with anti Ki67 antibody. (a-g) Immuno-reactivity against anti-caspase-3 antibody where, (a) G1 showed completely negative expression against anti-caspase-3 antibody within the hepatic parenchyma. (b,c) G2 showed diffuse positive immuno- localizations against anti-caspase-3 antibody (arrowhead) that were widely expressed in almost of the hepatic parenchyma confirming widespread of apoptosis. (d) G3 showed moderate immuno-reactivity against anti-caspase-3 antibody (arrow head) where groups of positively reacted hepatocytes were collected as a patches within the hepatic parenchyma. (e) G4 showed mild immuno-localization against anti-caspase-3 antibody (arrow head) where only individual positively reacted hepatocytes where distinguished within the negatively reacted hepatic parenchyma. (f) G5 showed completely negative expression against anti-caspase-3 antibody within the hepatic parenchyma in all of the examined sections. (g) G6 showed mild immuno-localization against anti-caspase-3 antibody (arrow head) where only individual positively reacted hepatocytes where distinguished within the negatively reacted hepatic parenchyma. (h,i) Immuno-reactivity against anti-Ki67 antibody where, G6 showed strongly up regulations of anti-Ki67 expression (arrowhead) that were widely expressed in almost of the hepatic parenchyma confirming widespread of the proliferated hepatocytes. Stain: a-g) Immunohistochemical stain against anti-caspase-3 antibody, (h,i) against anti-Ki67 antibody. Scale bars: All = 30 µm, except a = 200 µm

 Concerning immunohistochemical reactivity of liver against anti-caspase-3 antibody in G1-6, in addition anti-Ki67 antibody in G6 where, the positive signal is mostly expressed in the nuclei of morphologically identifiable apoptotic cells when stained with anti-caspase-3 antibody and mostly expressed in the proliferated cells when stained with anti Ki67 antibody. The expression of caspase-3 in different groups were diameter dependent; the smaller diameters of GNPs the more up regulations of the caspase-3 where G1 exhibited completely negative expression against anti-caspase-3 antibody within the hepatic parenchyma (Figure 11a), G2 showed diffuse positive immuno- localizations against anti-caspase-3 antibody that were widely expressed in almost of the hepatic parenchyma confirming widespread of apoptosis (Figure 11b and 11c).Furthermore,G3noticed moderate immuno-reactivity against anti-caspase-3 antibody where groups of positively reacted hepatocytes were collected as a patches within the hepatic parenchyma (Figure 11d).G4showed mild immuno-localization against anti-caspase-3 antibody where only individual hepatocytes that reacted positively where distinguished within the negatively reacted hepatic parenchyma (Figure 11e). G5 showed completely negative expression against anti-caspase-3 antibody within the hepatic parenchyma in all of the examined sections (Figure 11f). Moreover, G6clarified mild immuno-localization against anti-caspase-3 antibody where only individual positively reacted hepatocytes were observed within the negatively reacted hepatic parenchyma (Figure 11g). But, with anti-Ki67 antibody, G6 exhibited strongly up regulations of anti-Ki67 expression that were widely expressed in almost of the hepatic parenchyma in almost of the examined sections, confirming widespread of the proliferated hepatocytes (Figure 11h and 11i).

## Discussion

 Nanoparticles have novel properties, and thus, can offer great possibilities for the environment and human diseases. Some toxicological studies clarified that small-sized GNPs have harmful effects on the health of humans and living species in the environment. It has been reported that exposure to smaller sized GNPs produced more inflammatory and cytotoxic reactions when compared with exposure to larger sized GNPs of same mass concentration due to their highly reactive role, surface area, and number of NPs within the biological tissues.^34,35^

 The NPs can interact with the biological system and had negative consequences. One of these negative impacts might be a disruption in the normal balance of oxidative stress and antioxidant defense indices, which can lead to a variety of pathogenic repercussions. Oxidative stress has been recognized as a potential mechanism of nanoparticle toxicity.^36^

 Our investigation revealed that all tested antioxidant enzymes; GPX, SOD & CAT clarified a significant decrease in all groups injected with different diameters of GNPs; 10, 20 & 50 nm but the G2 injected with 10 nm was the most affected group and recorded the most decrease of all enzymes levels. These results were similar to that reported by Negahdaryet al^37^ who clarified that the concentration of SOD, CAT & GPX in the serum were decrease after the administration of GNPs on male mice. Regarding the effect of Qur, our results were supported by the findings of Abdelhalim et al^31^ who observed that Qur and arginine are highly potent antioxidants that have the ability to protect hepatocytes from damage, which is produced by oxidative stress and the associated vascular problems caused by the generation of GNPs, from the production of ROS.

 A significant elevation of AST, ALT & ALP were recorded in all groups injected with different diameters of GNPs but the G2 was the most affected group and recorded the highest level. This result is in agreement with Orabi et al^30^ who reported that GNPs lead to sharp increasing in AST, ALT & ALP levels in 10 nm but 50 nm had no significant effect on serum AST, ALT & ALP level. In addition, GNPs lead to significant increase in AST, ALT & ALP in general.^31^ But with Qur, our result revealed that the G5 had a value near to the control group and G6 had a good impact and recorded a significant decrease in AST, ALT & ALP levels nearing to the control group. These results are in coinciding with^31^ who claimed that AST, ALT and ALP levels were decreased in the treated groups with Qur.

 The present study demonstrated a significant decrease of albumin, in all injected groups with GNPs but recorded lower level in G2 followed by G3 then G4. These results are similar to ^38^ who reported that albumin level on 5nm and 10 nm decreased more than 30 nm & 60nm.

 The present study clarified a significant elevation of bilirubin (total, direct, indirect) in all injected groups with GNPs and was more in G2. But with Qur, G6 showed decrease in bilirubin level when compared to G2. Recent study reported that total bilirubin increased with different diameters of GNPs but in 5 nm & 10 nm more than 30 nm & 60 nm and decreased with Qur when compared with the control group.^38^ And also, other studyclarified that total bilirubin increased in all injected groups with GNPs. In addition, they added that Qur and arginine had a significant decreased in liver enzymes and the oxidative stress, therefore improving the liver damage and hepatotoxicity induced by GNPs.^31^

 The current study announced a significant decrease of hemoglobin level in G2 injected with 10 nm GNPs. This result was in agreement with Ramadhan and Ghareeb^39^ who distinguished that a very significant decrease in HB, RBCs in groups that take GNPs. Meanwhile, G6 demonstrated a good value and recorded elevation in HBs towards the normal level when compared with G2. These improvements indicate that the Qur possess a potent protective action on the haemopoietic tissue.

 Meanwhile, WBCs recorded a significant elevation in G2 & G3 but G2 was the most affected group that recorded the highest elevation between the injected groups. These results go hand in hand with Ramadhan and Ghareeb^39^ who noticed a significant increase in WBC levels when injected with GNPs. Our data was also supported by Morsy et al^40^ who found that 10 nm GNPs elevate the WBC count more than the corresponding controls, whereas HB was markedly decreased.

 RBCs and platelets were clarified non-significant changes between all groups. These finding was the same as the findings of Orabi et al^30^ who mentioned that 10 & 50 nm GNPs had no effect on platelets count when compared to the control group.

 The histopathological examination of the liver of G2 clarified diffuse acute cellular swelling due to hydropic degenerations (ballooning degeneration) in the hepatic parenchyma characterized with enlarged, swollen hepatocytes, with small pyknotic nuclei and pale cytoplasm with eosinophilic depress with general disorganization of the hepatic cords. These results were in agreement with Elbehiry et al^41^ who declared that the hepatocytes in the liver showed severe hydropic degeneration and necrosis after being exposed to GNPs at a dosage of 10 nm for 30 days. In addition, recent study described cloudy swelling exhibited as a result of disturbances of membrane function that lead to a massive influx of water and Na^+^ due to GNPs effects. Cellular swelling might be accompanied by leakage of lysosomal hydrolytic enzymes that lead to cytoplasmic degeneration and macromolecular crowding. Hydropic degeneration was a result of ion and fluid homeostasis that leads to an increase in intracellular water. The vacuolated swelling of the cytoplasm of the hepatocytes of the GNPs treated rats might indicate acute and subacute liver injury induced by these NPs.^4^

 G2 clarified numerous pleomorphic nuclei with variable size, shape and activity within the hepatocytes. In addition, some nuclear changes were also obvious in the degenerated hepatocytes as karyorrhexis & karyolysis. Also, single coagulative necrotic cells were distinguished in between the hydropic degenerated hepatocytes and characterized with ghostly appearance, enlarged cells and eosinophilic cytoplasm. The results agree with Abdelhalim and Jarrar^4^ who clarified that 10 nm of GNPs has effects on liver tissue more than 20 nm and 50 nm and indicate that 10 nm produces sporadic spotty well-defined necrosis in some hepatocytes. This alteration was detected in the liver of rats exposed to 10 nm size particles and to a lesser extent with 20 nm particles, but was not seen with those exposed to 50 nm size particles. The observed hepatocyte necrosis due to GNPs exposure might indicate oxidative stress on these cells by glutathione depletion. And also, other study declared severe hydropic degeneration and necrosis of the hepatocytes after exposed to 10 nm GNPs for 30 days.^41^

 Our findings announced sever steatosis; fatty changes, micro & macrovascular fat droplets aggregations within the hepatocyte’s cytoplasm of variable shape and size. These findings were in parallelism with^4^ who mentioned fatty change in some swelling hepatocytes of rats exposed to 10 nm GNPs and to lesser extent in the ones exposed to larger particles and described that the seen hepatocytes abnormal retention of lipids in the present investigation induced by GNPs might indicate toxic injury to liver in the form of hepatocytes liposis by these particles.

 G2 noticed sever inflammatory cells infiltrations of mainly lymphocytes surrounding sever dilated & congested blood vessels within the portal triad. This result was in agreement with Cho et al^42^ who reported acute liver inflammation and apoptosis after being injected with 13 nm sized GNPs. In addition, they found a significant increase in the expression of pro-inflammatory cytokines in organs. Also, these results go hand in hand with Abdelhalim and Jarrar^4^ who noticed occasional inflammatory cells infiltration in the portal triads. The infiltrate cells were mainly lymphocytes and plasma cells.

 With Perl’s Prussian blue stain, G2 clarified diffuse hemosiderosis within almost hepatocytes cytoplasm. This result was in parallelism with Eldin et al^43^ who demonstrated that the hepatocytes and Kupffer cells contain hemosiderin, which can be seen as blue granules when stained with Prussian blue iron. In addition, with PAS stain, very pale hepatic parenchyma was also identified as a result of diffuse glycogen depletions within the hepatocytes cytoplasm. These results close and similar to the finding that reported by Almansour et al^44^ who reported partial glycogen depletion in the hepatocytes of animals received 10 nm GNPs for 10 days and to lesser extent in those exposed to 20 nm GNPs. And they added that the depletion was more prominent in the hepatocytes surrounding the pericentral areas while those surrounding the portal spaces were less affected in male rats.

 Resembling other metal oxide NPs, we observed that several hepatic histopathological changes induced after the injection of GNPs in the present study were typically similar to that clarified after the injection of copper oxide NPs as diffuse hydropic degenerations of hepatocytes, severe hepatic sinusoidal congestion, necrosis, sever steatosis, diffuse hemosiderosis and diffuse glycogen depletions within the hepatocyte’s cytoplasm.^45^

 Meanwhile, the liver of G3 that injected intra peritoneal with GNPs in a dose of 20 nm showed moderate hydropic degeneration of the hepatocytes, moderate blood vessels dilatation with moderate congestions in portal triad, moderate periductal inflammatory cells infiltrations, with moderate inflammatory cells infiltrations in the portal triad surrounding portal blood vessels, moderate fibrous connective tissue proliferations of mostly collagen fibers surrounding the portal blood vessels with moderate sinusoidal dilatation & congestion in between the hepatocytes. These findings were in agreement with Fadia et al^46^ who observed congestive vessel and dilated central vein in the rat liver after 20 nm treatment with GNPs, as well as lymphocyte infiltration, inflammation, and isolated regions of necrosis. Furthermore,recent investigation clarified cell necrosis and leukocyte infiltration in the hepatic parenchyma in group received 20 nm GNPs after 24 hours of treatment.^47^ Moreover, other results confirmed that the pathological changes in G3 injected with 20nm were less than that observed in G2 injected with 10nm GNPs.^4^

 Meanwhile, G4 received 50 nm GNPs showed normal, intact hepatic parenchyma, with obvious normal organized hepatic rays looks like that in the control groups but with slightly pathological changes as slight hydropic degeneration of the hepatocytes, mild dilatation & congestion of central vein, with mild inflammatory cells infiltrations in between the hepatocytes. These results were completely going hand with Barathmanikanth et al^48^ who showed that the shape of the hepatocytes in the liver treated with 50 nm of GNPs for 15 days was not very different from the shape of the hepatocytes in the control group. Moreover, our results were supported by Ibrahim et al^49^ who mentioned that GNPs of 50 nm diameter produced comparatively less pathological changes in the liver when compared to the 5nm GNPs as generally the larger diameters of GNPs, the lesser toxicity for the hepatic tissue.

 G6 treated orally with Qur for along 14 days then injected intra peritoneal with gold NPs in a dose of 10 nm showed a significant good result when compared with G2 where the hepatic parenchyma was appeared looks like normal with normal tissue architecture & cellular details without any abnormalities; intact hepatocytes, vasculature and obvious hepatic cords. These results are in coincidence with Liu et al^50^ who clarified that the Qur improved the liver after injury with NPs and also, reported that hepatic-injured rats when treated with Qur could reduce hepatic injury and protect hepatocytes against damage. In addition, they also clarified that Qur prevents the development of hepatic fibrosis and reduces toxicant-induced liver injury. These findings were supported by Abdelrahman et al^51^ who noticed that the Qur has more potent cytoprotective, anti-inflammatory, and anti-apoptotic effects and has a significant mitigating role against the GNPs toxicity. And also, the position of Qur was supported by Eldin et al^43^ who demonstrated that histopathological effects induced by the metal particle were observed to be mitigated by concurrent Qur administration, as evidenced by a lack of Prussian blue staining in the Qur-treated group. So, the current study can confirm the potent effect and excellent value of Qur as a hepato-protective substance against the hepatotoxicity induced by GNPs.

 Finally, the current study clarified that the greatest pathological changes were noticed in G2 received 10 nm GNPs more than other groups that received larger diameters; 20 and 50 nm GNPs. Where, this finding was confirmed by the result of Abdelrahman et al^51^ who clarified that the size of GNPs is pivotal in their pathological effect on the renal tissues where the small-sized GNPs, 10 nm have more potent cytotoxic, inflammatory, and apoptotic effects rather than the larger ones.

## Conclusion

 The current study can conclude that the smaller diameters of GNPs induce a potential oxidative stress, cytotoxic effect, sever histopathological damages and apoptosis in the hepatic tissues which was confirmed by a significant decrease of the antioxidant enzymes, with a significant elevation of most liver enzymes and some hematological parameters in the group treated with 10 nm GNPs but with increasing the diameter of GNPs, the treated groups with 50 nm clarified less hepatic toxicity. In addition, Qur demonstrated a significant prophylactic role against the hepatotoxicity induced by GNPs.

## Acknowledgments

 We would like to express deep appreciation to the administration of the Faculty of Veterinary Medicine, Zagazig University for allowing us to use all laboratories, facilities, and chemicals needed for the accomplishment of this work.

## Competing Interests

 The authors declare that they have no competing interests.

## Ethical Approval

 The research protocol has been reviewed and approved by the Institutional Animal Care and Use Committee, Zagazig University, Egypt, with an approval number (ZU—IACUC; No. ZU-IACUC/2/F/250/2022).

## Funding

 This work did not receive any grant from any commercial, public or not-for-profit sectors.
